# A New Perspective on the Role of Glutamine Synthetase in Nitrogen Remobilization in Wheat (*Triticum aestivum* L.)

**DOI:** 10.3390/ijms222011083

**Published:** 2021-10-14

**Authors:** Yihao Wei, Lulu Wang, Butan Qin, Huiqiang Li, Xiaoran Wang, Zhiyong Zhang, Xiaobo Zhu, Xinming Ma, Xiaochun Wang

**Affiliations:** 1 Collaborative Innovation Center of Henan Grain Crops, College of Agronomy, Henan Agricultural University, Zhengzhou 450000, China; yihaowei@stu.henau.edu.cn (Y.W.); wll9501@stu.henau.edu.cn (L.W.); qinbutan@stu.henau.edu.cn (B.Q.); zhiyongzhang@henau.edu.cn (Z.Z.); maxinming@henau.edu.cn (X.M.); 2 Department of Biochemistry and Molecular Biology, College of Life Science, Henan Agricultural University, Zhengzhou 450000, China; huiqiangli@henau.edu.cn (H.L.); xiaoranwang@henau.edu.cn (X.W.); xiaobo2021@stu.henau.edu.cn (X.Z.)

**Keywords:** nitrogen use efficiency, grain filling, phloem, nucleic acid, ureides, degradation, ammonium assimilation

## Abstract

Glutamine synthetase (GS), a key enzyme in plant nitrogen metabolism, is closely related to nitrogen remobilization. However, how GS isoforms participate in nitrogen remobilization remains unclear. Here, the spatiotemporal expression of the *TaGS* gene family after anthesis was investigated, and the results showed that TaGS1;1 was mainly encoded by *TaGS1;1-6A*, while the other isozymes were mainly encoded by *TaGS* localized on the A and D subgenomes. *TaGS1;2-4A/4D* had the highest expression level, especially in rachis and peduncle. Furthermore, immunofluorescence showed TaGS1;2 was located in the phloem of rachis and peduncle. GUS (β-glucuronidase) staining confirmed that *ProTaGS1;2-4A/4D::GUS* activity was mainly present in the vascular system of leaves, roots, and petal of *Arabidopsis*. Ureides, an important transport form of nitrogen, were mainly synthesized in flag leaves and transported to grains through the phloem of peduncle and rachis during grain filling. *TaAAH*, which encodes the enzyme that degrades ureides to release NH_4_^+^, had a higher expression in rachis and peduncle and was synchronized with the increase in NH_4_^+^ concentration in phloem, indicating that NH_4_^+^ in phloem is from ureide degradation. Taking the above into account, TaGS1;2, which is highly expressed in the phloem of peduncle and rachis, may participate in N remobilization by assimilating NH_4_^+^ released from ureide degradation.

## 1. Introduction

Wheat (*Triticum aestivum* L.) is one of the most important cereal crops cultivated worldwide and is mainly used for human consumption [1]. Nitrogen (N) is a key limiting factor for the yield and quality of wheat [2,3]. However, excessive application of nitrogen fertilizer not only causes economic losses but also leads to environmental pollution, which is a stumbling block to the sustainable development of agriculture. Improving nitrogen use efficiency is an effective strategy to remove this obstacle [3,4,5,6].

Nitrogen utilization of wheat includes nitrogen absorption, assimilation, translocation, and remobilization. Efficient N remobilization during grain filling is crucial to improve nitrogen use efficiency [7,8]. In wheat, 60% to 95% of grain N at harvest comes from the remobilization of N stored before anthesis. The glutamine synthetase (GS; EC 6.3.1.2) activity is highly correlated with the amount of N remobilized and the grain yield [2,8,9,10,11,12].

GS catalyzes the ATP-dependent fixation of ammonium (NH_4_^+^) to the glutamate (Glu) to form glutamine (Gln) [13]. According to subcellular location, plant GS is classified into two groups: the cytosolic glutamine synthetase (GS1) and the chloroplast glutamine synthetase (GS2) [14]. In diploid plants, such as *Arabidopsis*, rice, and maize, GS1 is encoded by 3–5 nuclear genes, while GS2 is encoded by a single nuclear gene [15]. In hexaploid wheat (2n = 42, AABBDD genomes), TaGS1 is encoded by nine nuclear genes, while TaGS2 is encoded by three nuclear genes [16]. According to phylogenetic tree analysis of GS isoforms of cereals, the *TaGS* gene family is classified into four subfamilies: *TaGS1;1-6A/6B/6D*, *TaGS1;2-4A/4B/4D*, *TaGS1;3-4A/4B/4D,* and *TaGS2-2A/2B/2D* [15]. Recent studies have mainly focused on the functional differences between TaGS1;1, TaGS1;2, TaGS1;3, and TaGS2 [17,18,19].

TaGS2, located in chloroplasts, is the predominant isozyme in green tissue, and its dominating role is in the reassimilation of NH_4_^+^ released by photorespiration and assimilation of NH_4_^+^ derived from NO_3_^−^ reduction [20,21]. TaGS1;3, located in the pericycle and endodermis of roots, is mainly involved in the assimilation of NH_4_^+^ absorbed by roots. TaGS1;3 is also located in the aleurone layer and endosperm transfer cells of grains, which may play a key role in transporting Gln into the endosperm for gluten synthesis [17]. In flag leaves, the *TaGS1;1* transcript is located in the perifascicular sheath cell and *TaGS1;2* transcript is present in the vascular phloem cells and the parenchyma cells close to the xylem [18]. They are involved in N remobilization by assimilating NH_4_^+^ from protein turnover during leaf senescence [15,18]. However, TaGS1;1 and TaGS1;2 proteins are located in the mesophyll cells and xylem of leaves, respectively [19]. The differences in localization at the protein and mRNA levels highlight the importance of studying TaGS protein localization. Moreover, the transcripts of *TaGS1;1* and *TaGS1;2* are also located in the parenchyma cells near the vascular tissue and vascular cells of stem and rachis [18]. However, the relationship between TaGS and nitrogen remobilization in these organs is not clear.

Nitrogen remobilization mainly occurs during tissue senescence. During the grain filling of wheat, N-containing organics in the senescence source organs (leaves) are degraded into small molecules in order to be transported into the sink organs (grains) through the vascular tissue of peduncle and rachis [7,22,23,24,25,26,27]. Sink organs generally display little need for xylem due to their low transpiration rates, so N partitioning from the source leaves to sinks occurs in the phloem [28]. Protein and nucleic acid in flag leaves are important sources of organic nitrogen in preanthesis wheat [27,29]. During flag leaf senescence, the amino acids generated during protein turnover are transferred to grains via phloem, mainly in the form of Gln and alanine (Ala) [7]. Among the degradation products of nucleic acids, purine has the highest nitrogen content, and its degradation product, ureides, is one of the most important forms of transport for organic nitrogen. Ureide degradation can release four molecules of NH_4_^+^ [26], which are likely to be reassimilated by GS into Gln. During the transportation of ureides from the source organs to grains via the peduncle and rachis, if ureides is degraded and releases NH_4_^+^, then this part of NH_4_^+^ assimilation may be related to TaGS in the peduncle and rachis.

Not only do the four TaGS isozymes have different functions but the three *TaGS* genes in the same subfamily also have different functions [30,31,32]. Quantitative trait locus (QTL) analyses in wheat has shown that the QTL for GS activity of flag leaves only colocalizes with *TaGS2-2A* and *TaGS1;2-4A* among the 12 *TaGS* genes [32], and only *TaGS2-2B*, *TaGS1;2-4A*, and *TaGS1;3-4A* are associated with the QTL for grain protein content [30,31,32]. The promoter elements of three *TaGS* genes in the same subfamily are different [16], which may indicate that the *TaGS* genes in the same subfamily have different expression patterns and regulation mechanisms. Therefore, investigating the promoter activity of the 12 *TaGS* genes, detecting the spatiotemporal expression of the *TaGS* gene family, and analyzing ureide metabolism during the grain filling of wheat will help us elucidate how TaGS isozymes participate in N remobilization by NH_4_^+^ assimilation. Here, we discovered that TaGS1;2, located in the phloem of peduncle and rachis, may participate in nitrogen remobilization by assimilating NH_4_^+^ released from ureide degradation.

## 2. Results

### 2.1. The Spatiotemporal Expression Profile of TaGS Gene Family

**Figure 1:**
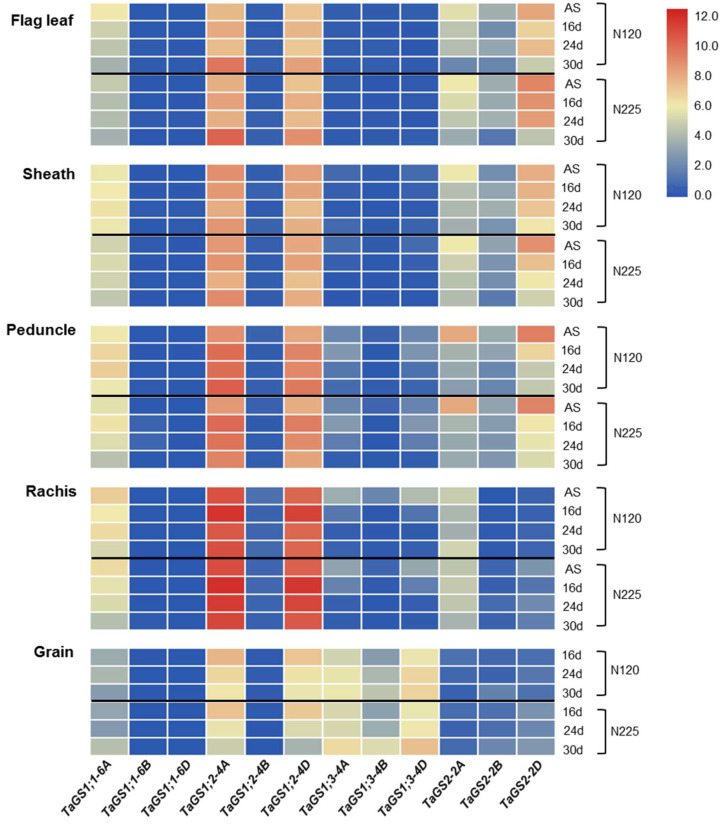
Heatmap of *TaGS* gene expression profiles in different organs in relation to N fertilization and development by qRT-PCR. The geometric mean of C_t_ values of *TaATPases* and *TaTEF* served to normalize the expression ratio for each gene. Data are means of six independent biological replicates. Gene expression data are shown as log_2_-transformed data of normalized data+1. AS, anthesis stage; 16 d, 24 d, and 30 d represent 16, 24, and 30 days after anthesis (DAA), respectively; N120, 120 kg N ha^−1^; N225, 225 kg N ha^−1^.

In order to investigate the function of TaGS in nitrogen remobilization from source (flag leaf) to sink (grain) through peduncle and rachis, the spatiotemporal expression patterns of the *TaGS* gene family during grain filling were explored by qRT-PCR. The results showed that *TaGS1;1-6A* was the major gene encoding TaGS1;1, and TaGS1;2 was mainly encoded by *TaGS1;2-4A* and *TaGS1;2-4D. TaGS1;2-4A/4D* had the highest expression level in the *TaGS* gene family. The genes encoding TaGS1;3 and TaGS2 were localized on the A, B, and D subgenomes, and the genes located in the D and A subgenomes had a higher expression (Figure 1). Under different nitrogen application conditions, most *TaGS* genes expressed did not change significantly except that *TaGS1;2-4A/4D* in rachis and *TaGS*2-*2A/2D* in flag leaves had a higher expression level under N225 than N120 (Figure 1).

There were significant differences in the expression levels of *TaGS* genes in different organs. *TaGS1;1-6A* was extensively expressed in flag leaf, sheath, peduncle, rachis, and grain; *TaGS1;2-4A* and *TaGS1;2-4D* were mainly expressed in peduncle and rachis; *TaGS1;3-4A/4B/4D* were mainly expressed in grain; and *TaGS2-2A/2B/2D* were mainly expressed in flag leaf (Figure 1). With the senescing of flag leaves, the expression levels of *TaGS1;1-6A* in flag leaves gradually decreased, while the expression levels of *TaGS1;2-4A/4D* gradually increased (Figure 1), suggesting that TaGS1;2 may play a more important role in nitrogen remobilization.

### 2.2. Promoter Activities of TaGS Gene Family

**Figure 2:**
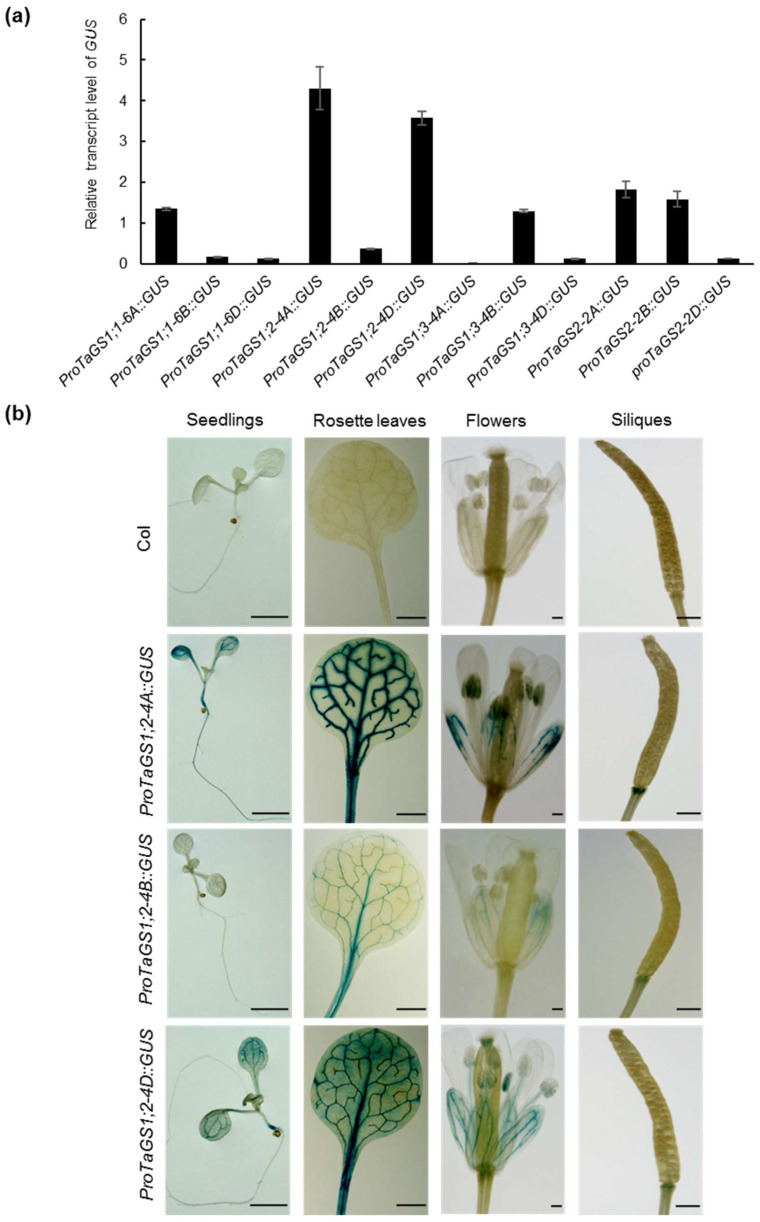
Patterns of *GUS* expression driven by the promoters of the *TaGS* genes in *Arabidopsis*. Transgenic lines were transformed with transcriptional fusions between the *TaGS* gene promoter and the GUS reporter gene. (**a**) qRT-PCR analysis of *GUS* expression in transgenic lines. Gene expression levels were normalized to reference gene *AtUBQ11*. Data are means of three independent biological replicates ± SD. (**b**) GUS staining was observed in 10 d old seedlings, rosettes leaves of 25 d old plants, and flowers and siliques of 40 d old plants. The blue signal indicates GUS activity. Scale bars are 1 mm.

To investigate the reasons for differential expression of the *TaGS* gene family, the expression patterns of *GUS* driven by the promoters of 12 *TaGS* genes in transgenic A*rabidopsis* were analyzed. By comparing the expression level of *GUS*, the activity of *ProTaGS1;1-6A/6B/6D* and *ProTaGS1;2-4A/4B/4D* were consistent with the expression trend of their corresponding genes in wheat. *ProTaGS1;1-6A* had a much higher activity than *ProTaGS1;1-6B/6D*. The expression level of *GUS* controlled by *ProTaGS1;2-4A/4D* was tenfold that of *ProTaGS1;2-4B*, the two most active promoters of the *TaGS* gene family (Figure 2a). However, the activity of *ProTaGS1;3-4A/4B/4D* and *ProTaGS2-2A/2B/2D* was not consistent with the expression level of their corresponding genes in wheat. The activity of *ProTaGS1;3-4B* was much higher than that of *ProTaGS1;3-4A/4D*, while *ProTaGS2-2A/2B* was approximately 15 times more active than *ProTaGS2-2D* (Figure 2a).

The blue signal was detected in the *ProTaGS1;2-4A/4B/4D::GUS* transgenic plants and was mainly present in the vascular tissues of the roots, hypocotyl, leaves, flowers, and the bottom of the siliques (Figure 2b). No obvious GUS activity was detected in the other transgenic plants. In addition, there were significant differences in the activities of the three promoters. GUS activity controlled by *ProTaGS1;2-4A/4D* was significantly higher than that controlled by *ProTaGS1;2-4B* (Figure 2b), consistent with the expression level of *TaGS1;2-4A/4B/4D* in wheat (Figure 1).

### 2.3. TaGS1;2 Located in Phloem of Peduncle and Rachis Participates in N Remobilization

**Figure 3:**
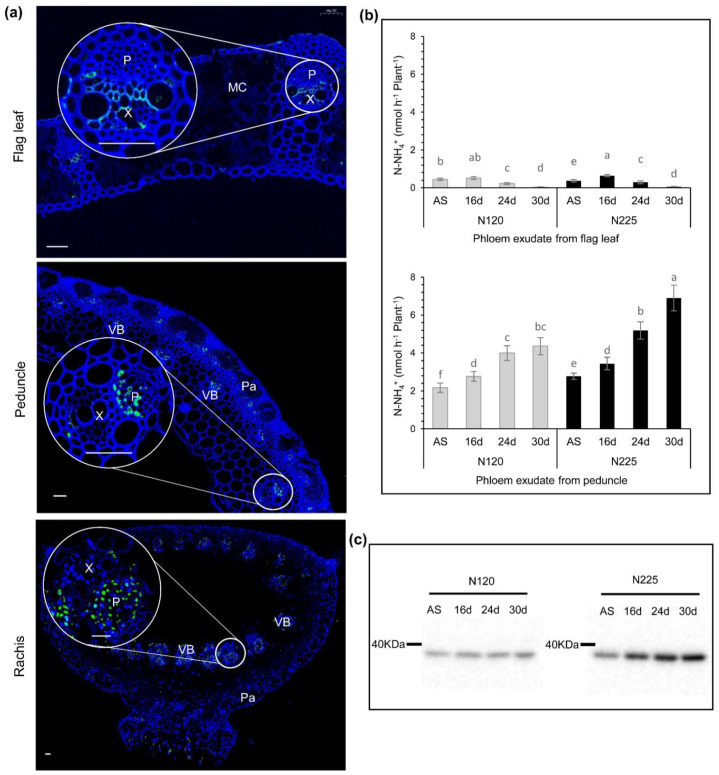
TaGS1;2 involved in nitrogen remobilization by NH_4_^+^ assimilation in the phloem of wheat. (**a**) Histological immunolocalization of TaGS1;2 in flag leaf, peduncle, and rachis transverse section. P, phloem; X, xylem; VB, vascular bundle; Pa, parenchyma. Bars = 50 μm. (**b**) The content of NH_4_^+^ in the phloem exudate of flag leaf and peduncle during grain filling. Data are the means of six independent biological replicates ± SD. The different letters above each sample indicate statistically significant differences, where *p* < 0.05 according to one-way ANOVA and Duncan post-hoc test. (**c**) Western blot analysis of expression profile of subunits in rachis during grain filling grown under N120 and N225 conditions. A total of 5 µg of soluble proteins extracted from the rachis was loaded in each lane.

*GUS,* driven by *ProTaGS1;2-4A/4B/4D,* is widely expressed in vascular tissues of *Arabidopsis*, indicating that TaGS1;2 may be related to nitrogen transport. Using specific antibodies of TaGS1;2 as a probe, the localization of TaGS1;2 in the flag leaf, peduncle, and rachis of wheat was studied by immunofluorescence. The results showed that TaGS1;2 was mainly located in the vascular tissues of wheat, including the xylem of flag leaf and the phloem of peduncle and rachis (Figure 3a). During grain filling, nutrients from the source organs are mainly transported to the grain through the phloem. Therefore, TaGS1;2 located in the phloem may play an important role in the transportation of nitrogen to the grain.

During grain filling, NH_4_^+^ content in the phloem exudate of flag leaf was extremely low, but the content of NH_4_^+^ in the phloem exudate of peduncle was very high. An increase in nitrogen application could significantly increase the NH_4_^+^ content (Figure 3b). Under the condition of N225, the content of NH_4_^+^ in the phloem exudate of peduncle was about 100 times that in the phloem exudate of flag leaf (Figure 3b). TaGS1;2 located in the phloem of peduncle and rachis that transport large NH_4_^+^ and the expression of TaGS1;2 in rachis increased with the increase in NH_4_^+^ content in phloem, and the expression level of TaGS1;2 under N225 was higher than that under N120 (Figure 3c). The high expression of TaGS1;2 in the phloem of rachis coincided with the high NH_4_^+^ export, suggesting that TaGS1;2 assimilates NH_4_^+^ in the phloem into Gln and is involved in the transportation of nitrogen to grain.

### 2.4. The NH_4_^+^ in Phloem of Peduncle and Rachis Is from the Degradation of Ureides

**Figure 4:**
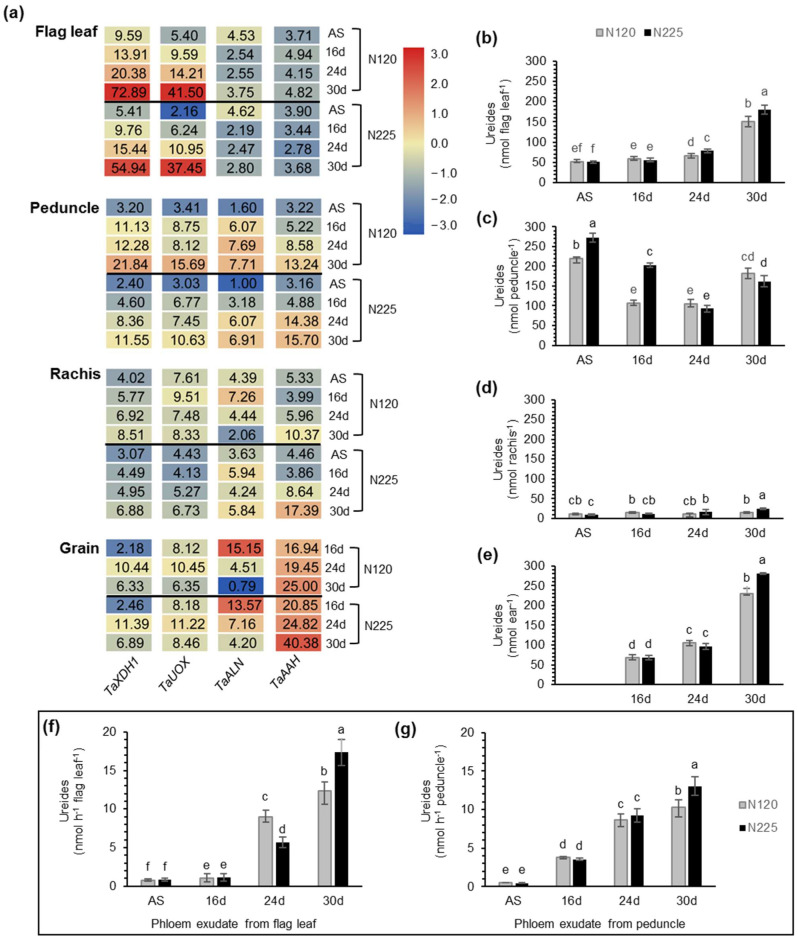
Characteristics of synthesis, transport, and degradation of ureides in wheat at the grain filling stage. (**a**) qRT-PCR analysis of the ureide metabolism genes in different organs in relation to N fertilization and development. The geometric mean of C_t_ values of *TaATPases* and *TaTEF* served to normalize the expression ratio for each gene. Data in the figure are origin data and the means of six independent biological replicates. Gene expression data are shown as log_2_-transformed data of normalized data+1. Ureides accumulation in the flag leaf (**b**), peduncle (**c**), rachis (**d**), and ear (**e**) during grain filling grown under N120 and N225 conditions. The content of ureides in the phloem exudate of flag leaf (**f**) and peduncle (**g**) during grain filling grown under N120 and N225 conditions. Data are means of six independent biological replicates ± SD. The different letters above each sample indicate statistically significant differences, where *p* < 0.05 according to one-way ANOVA and Duncan post-hoc test.

In order to investigate the sources of NH_4_^+^ in phloem, ureide metabolism during the grain filling of wheat was analyzed. Ureides includes allantoin and allantoate [33]. Xanthine dehydrogenase (TaXDH1) and urate oxidase (TaUOX), which are involved in wheat allantoin biosynthesis [27], were mainly expressed in flag leaves and significantly increased in the late stage of grain filling (Figure 4a). Ureides was mainly synthesized in flag leaves and then exported to the peduncle via the phloem, and the export of ureides reached the maximum at 30 DAA (Figure 4b,f). These results show that the synthesis and export of ureides mainly occurred during flag leaf senescence.

During the early grain filling stage, ureides accumulated in the peduncle (Figure 4c), and the export of ureides from peduncle to grain was lower (Figure 4g). With the development of grains, the rate of ureides export from peduncle increased significantly (Figure 4g), and the grains gradually became the main storage organ for ureides (Figure 4e).

Allantoin is metabolized into allantoate by allantoinas allantoinase (TaALN), and allantoate amidohydrolase (TaAAH) catalyzes the degradation of allantoate to produce NH_4_^+^ [27]. During the late grain-filling stage, the expression levels of *TaALN* and *TaAAH* in flag leaves were significantly lower than those in the peduncle, rachis, and grain (Figure 4a). Especially under N225 conditions, the expression level of *TaAAH* in grains at 30 DAA was about 10 times that in flag leaves (Figure 4a). The results indicate that the degradation of ureides mainly occurs in the peduncle and rachis that transports ureides and in the grains that stores ureides. Compared with N120, *TaAAH* had higher expression levels in peduncle, rachis, and grain under N225, and the corresponding NH_4_^+^ content in the phloem was higher (Figure 3b and Figure 4a). The phloem of rachis is an important channel for ureides to enter the grain. In rachis, the synthesis and accumulation of ureides was extremely low (Figure 4a,d), but the expression of *TaAAH* was high (Figure 4a). These results support the hypothesis that NH_4_^+^ in the phloem is from the degradation of ureides.

## 3. Discussion

In order to improve crop nitrogen use efficiency, GS has been studied numerous times owing to its essential role in N remobilization during grain filling [8,10,15]. The localization of GS1 in the vascular tissues and the developmental regulation of GS1 suggest that it is important for nitrogen remobilization [18,24]. Gene knock-out assay and QTL analyses in rice, maize, or wheat have shown that GS1 is necessary for grain filling [8,14,15,31,34]. In wheat, TaGS1 isozyme includes TaGS1;1, TaGS1;2, and TaGS1;3, but how they participate in N remobilization remains unclear.

### 3.1. TaGS1;2-4A/4D Were Highly Expressed in Rachis and Peduncle during Grain Filling

This study is the first to report that *TaGS1;2-4A/4D* had the highest expression level in the *TaGS* gene family, especially in rachis and peduncle during grain filling (Figure 1). Furthermore, *GUS* driven by the *TaGS* genes promoter had different transcriptional levels in *Arabidopsis*, among which *ProTaGS1;2-4A/4D* had the highest activity (Figure 2a). Only *ProTaGS1;2-4A/4B/4D::GUS* transgenic plants had GUS activity, and it was mainly distributed in the vascular tissues of the leaf, root, and petal (Figure 2b), which is consistent with the expression of *TaGS1;2-4A/4D* in wheat. Under natural light conditions, *TaGS2-2A/2D* had a high expression level in the flag leaves of wheat (Figure 1), but no GUS activity was detected in *ProTaGS2-2A/2B/2D::GUS* transgenic plants. However, after 1 h of natural light induction, GUS activity could be detected in *ProTaGS2-2A::GUS* transgenic plant (Figure S2), indicating that the activity of *ProTaGS2-2A* is regulated by light, which is consistent with our previous research [35].

### 3.2. TaGS1;2 Participates in N Remobilization by Assimilating NH_4_^+^ from Ureide Degradation

GS activity is highly correlated with the amount of N remobilized [10], and *GS1* overexpression can promote N remobilization from senescent leaves to developing ones in tobacco [36,37]. Glutamine synthetase is an enzyme, not a transporter or a carrier protein. How does GS participate in nitrogen remobilization? During leaf senescence, *TaGS1;1* and *TaGS1;2* fulfil a key function in N remobilization by assimilating NH_4_^+^ generated from protein degradation [18]. We found that TaGS1;2 and NH_4_^+^ coexisted in the phloem of rachis and peduncle (Figure 3a,b), and a large number of amino acids (mainly Gln) entered into grains through the phloem during grain filling [17]. These results suggest that NH_4_^+^ in the phloem may be assimilated into Gln by TaGS1;2. In the phloem, the content of Gln is much higher than that of Glu [7]. TaGS1;2 has a high affinity for Glu and can be significantly activated by Gln [17], which is helpful for Gln synthesis in the phloem.

Where is the NH_4_^+^ in phloem of peduncle and rachis from? The main sources of NH_4_^+^ in plants include direct absorption from soil by roots, NO_3_^−^ reduction, photorespiration, and the degradation of protein and nucleic acid [8,27]. The NH_4_^+^ absorbed by wheat from the soil is mainly directly assimilated into organic nitrogen by the roots and is rarely transported to the shoot in the form of NH_4_^+^ [19,38,39]. NH_4_^+^ from NO_3_^−^ reduction and photorespiration is directly assimilated into Gln by GS2 [8,15,35], and this part of NH_4_^+^ is not transported among organs. It has been reported that protein degradation produces NH_4_^+^ during leaf senescence [24,40], but we found that there was no obvious NH_4_^+^ export from the phloem of flag leaves during grain filling (Figure 3b). During flag leaf senescence, degraded proteins were mainly exported in the form of amino acids, such as Gln and alanine (Ala) [17].

Nucleic acid is also an important N-containing organic molecule in plant tissues [27]. The content of the total nucleic acid in wheat flag leaves is about 2660 nmol/g DW [26]. During tissue senescence, the N of nucleic acid is mainly transported in the form of ureides [27]. Ureides are synthesized mainly in the flag leaf, then exported from the phloem of flag leaf into grain through phloem of peduncle and rachis, and eventually accumulate in the grain (Figure 4), which is consistent with Casartelli et al., who found allantoin accumulation in the grain [27]. TaAAH catalyzes the ureide degradation and releases NH_4_^+^ [26]. The expression level of *TaAAH* was very low in flag leaves but high in grains, peduncle, and rachis (Figure 4a). The expression level of *TaAAH* in grains was about 4–10 times that in flag leaves (Figure 4a), and the content of NH_4_^+^ in grains is about five times that in flag leaves [17]. These results indicate that the high concentration of NH_4_^+^ in grains is related to degradation of ureides catalyzed by TaAAH.

The phloem of rachis is a major channel for ureides into grain. In rachis, the synthesis and accumulation of ureides was very low (Figure 4a,d), but the expression of *TaAAH* was high (Figure 4a), indicating that the ureides, during transportation, can be degraded by TaAAH. TaGS1;2, located in the phloem of rachis, can assimilate the NH_4_^+^ released from ureide degradation. TaGS1;2 located in the phloem of peduncle, may also have a similar function. However, this hypothesis requires further research using in vivo ^15^N labelling experiments.

### 3.3. TaGS1;2-4A Is a Candidate Gene for Improving Nitrogen Remobilization Efficiency in Wheat

During grain filling, the expression of *TaGS1;1* and *TaGS1;2* are increased continuously [18,24,41]. However, the QTL for flag leaves GS activity is not colocalized with *TaGS1;1* [32]. In this study, we found that the expression level of *TaGS1;1-6A* continued to decrease during grain filling, while the expression level of *TaGS1;2-4A/4D* continued to increase and was much higher than that of *TaGS1;1-6A* (Figure 1). In addition, the activity of *ProTaGS1;2-4A/4D* was much higher than *ProTaGS1;1-6A* in *Arabidopsis* (Figure 2a). These results suggest that *TaGS1;2-4A/4D* plays a more important role in the N remobilization in flag leaves.

In *Arabidopsis*, *AtGln1;1-5* are expressed in the phloem of vascular tissue. *AtGln1;1*, *AtGln1;2*, and *AtGln1;3* act together for N remobilization and seed filling [5]. In wheat, TaGS1;2 is widely distributed in the vascular tissues, especially in the phloem of peduncle, rachis (Figure 3a), and grain [17]. TaGS1;2 is mainly encoded by *TaGS1;2-4A* and *TaGS1;2-4D*, and *TaGS1;2-4A* has a higher expression level (Figure 1) and is closely related to QTL for GS activity in flag leaves and grain protein content [30,32], which suggests that *TaGS1;2-4A* may be a candidate gene for improving nitrogen remobilization efficiency.

**Figure 5:**
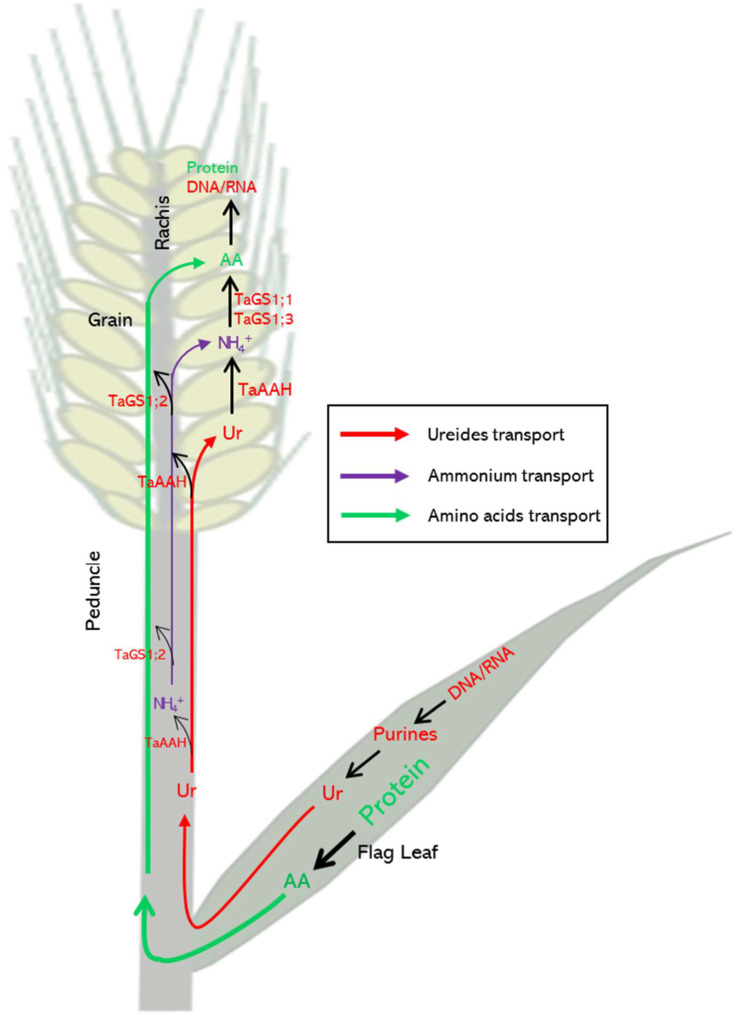
A schematic model of TaGS1;2 involved in the nitrogen remobilization pathway. The transport of ureides (Ur), NH_4_^+^, and amino acids (AA) is indicated by yellow, purple, and green arrows, respectively. Most of the ureides synthesized in flag leaves is transported to the grain via the phloem. During the transport, part of the ureides is degraded, and the released ammonium is assimilated into Gln by TaGS1;2, which is located in the phloem of the peduncle and rachis.

In summary, our current study revealed a novel perspective on the role of glutamine synthetase in nitrogen remobilization in wheat. TaGS1;2 was highly expressed in the peduncle and rachis, and its promoter activity was widely distributed in vascular tissues, indicating that it is the main TaGS isozyme in N remobilization during grain filling in wheat. TaGS1;2 encoded by *TaGS1;2-4A* and *TaGS1;2-4D* was located in the phloem of the peduncle and rachis, which may participate in nitrogen remobilization by assimilating NH_4_^+^ released from ureide degradation (Figure 5).

## 4. Materials and Methods

### 4.1. Wheat Growth Conditions and Experimental Design

The field experiment was conducted during the 2019–2020 growing season at the experimental station of Henan Agricultural University in Xuchang, Henan, China (113°48′34″ E, 34°7′56″ N). The Xuchang site is in the center of China and has a warm temperate continental monsoon climate. Meteorological data, including air temperature and rainfall, were acquired for growth seasons from the meteorological station of Xuchang (Table S1). The previous crop was maize. The soil contained 16.2 g organic matter kg^−1^, 1.30 g total N kg^−1^, 80.2 mg water-hydrolyzable N kg^−1^, 15.4 mg rapidly available phosphate kg^−1^, and 115.14 mg rapidly available potassium kg^−1^. Two nitrogen fertilizer levels of 120 and 225 Kg N ha^−1^ were set up, represented by N120 and N225, respectively. The experiment was conducted in a field plot with a size of 8 m × 2.4 m and repeated three times. Urea (46.4% N) was used as a nitrogenous fertilizer to perform the experiment. A total of 60% of the total N was directly mixed into the soil and combined with calcium superphosphate (120 kg ha^−1^) and potassium chloride (120 kg ha^−1^) before plowing, and 40% was dissolved in water and applied to the soil at the elongation stage. The seeds of Yumai 49 (YM49) were sown on 17 October 2019 in rows at 20 cm spacing with 12 rows per plot at a density of 290 plants m^−2^. YM49 is one of the popularized cultivars in the Henan province of China and has the characteristics of a high and stable yield and high nitrogen use efficiency [41]. YM49 seeds were purchased from Henan Pingan Seed Co., Ltd. (Jiaozuo, China). Irrigation was carried out in the overwintering stage and stem elongation stage.

The anthesis stage was defined as more than 50% of the spikes within a plot showing protruding anthers. To track days after anthesis, the stems with normal development and uniform size were labeled at anthesis. At the anthesis stage (AS) and 16, 24, and 30 days after anthesis (DAA), the flag leaf, sheath, peduncle and rachis, and grain of 10 stems were mixed, frozen in liquid N, and stored at −80 °C for the measurement of physiological indicators and gene expression. Two samples were collected from each of the three plots for a total of six replicates. The middle parts of the flag leaf, peduncle, and rachis from plants at 16 DAA were sampled and immediately immersed in fixative for the immunolocalization studies.

### 4.2. Quantitative Real-Time PCR

Total RNA was extracted from plant tissue using HiPure HP Plant RNA Kit B (Guangzhou Magen Biotechnology Co. Ltd., Guangzhou, China) according to the manufacturer’s protocols. cDNA was synthesized using the RT III Super Mix with dsDNase (Monad Biotech Co., Ltd., Shanghai, China). Quantitative real-time PCR (qRT-PCR) was performed on a Step One Real-Time PCR System (Life Technologies Corporation, Carlsbad, CA, USA) with SYBR Green qPCR Mix (Monad) for the assay. All primers (Sangon Biotech Co., Ltd., Shanghai, China) of the *TaGS* genes used for qRT-PCR analysis are shown in Table S2. The specificity of each pair was verified by melting curve analysis and sequencing of the products. According to Casartelli et al. [27], primers of *TaXDH1*, *TaUOX*, *TaALN*, and *TaAAH* were synthesized by Sangon. The primers are listed in Table S2. The qPCR mix was composed of 10 µL SYBR Green qPCR Mix (Monad), 5 µL diluted cDNA 1:20 (*v*/*v*), 0.5 µL forward primer and 10 µM reverse primer, and 4 µL of sterile nuclease-free water. The conditions for qRT-PCR were as follows: 95 °C for 10 min, 40 cycles of 95 °C for 15 s, 58 °C for 15 s, and 72 °C for 20 s. Fluorescence readings were taken during the elongation step (72 °C). Melting curves were obtained from 60 to 95 °C with a 0.5 °C increase every 15 s. The *TaATPases* (*Ta54227*) and *TaTEF* (*Ta53964*) were utilized as reference genes [42]. The geometric mean of C_t_ values of *TaATPases* and *TaTEF* served to normalize the expression ratio for each gene. The relative expression levels of genes were calculated via the 2^^(−ΔΔCt)^ method.

### 4.3. Binary Constructs and Plant Transformations

The wheat cultivar YM49 was used to isolate *TaGS* gene promoters. An amplification of approximately 2000 bp of each *TaGS* gene promoter was performed using the genomic DNA of wheat leaves as templates. Gene-specific primer couples are listed in Table S3. The amplified PCR products were cloned into a pTOPO vector (Aidlab Biotechnologies Co., Ltd., Beijing, China) and fully sequenced.

To construct the *ProTaGS:GUS* fusion gene, the promoters of each *TaGS* gene were amplified using these cloning vectors as templates. The primers are listed in Table S4. The PCR products were ligated into the binary vector pCAMBIA1301 between the *EcoR* I and *Nco* I sites. The recombinant vectors were constructed using ClonExpress One Step Cloning Kit (Vazyme Biotech Co., Ltd., Nanjing, China). The authenticity of the fusion constructs was confirmed by DNA sequencing, followed by transformation into *Agrobacterium* strain GV3101 for *Arabidopsis* stable transformation. Transgenic plants in the wild-type Columbia-0 background were obtained by floral dipping [43] and selected for their hygromycin resistance. The resistant seedlings were transferred to soil and verified by genomic PCR. More than six transgenic plants were obtained for each construct. Only three selected representative lines were used in all our experiments.

### 4.4. Expression Analysis of GUS in Transgenic Plants

Total RNA was extracted from transgenic *Arabidopsis* using HiPure HP Plant RNA Kit B (Magen). cDNA was synthesized using the RTIII Super Mix with dsDNase (Monad). Quantitative real-time PCR (qRT-PCR) was performed on a Step One Real-Time PCR System (Life) with SYBR Green qPCR Mix (Monad) for the assay. The primers are listed in Table S2. *AtUBQ11* were used as reference genes; relative expression levels of *GUS* were calculated via the 2^^(−ΔΔCt)^ method.

### 4.5. Histochemical GUS Staining Assay

Plants carrying the *ProTaGS::GUS* construct were incubated in GUS staining solution (50 mM KH_2_PO_4_, 50 mM K_2_HPO_4_, 10 mM Na_2_EDTA, 0.1% (*v*/*v*) Triton X-100, 0.5 mM ferricyanide, 0.5 mM ferrocyanide, and 20 mM 5-bromo-4-chloro-3-indolyl-β-D-glucuronic acid (X-Glc A)) at 37 °C for 12 h and then decolorized by 75% ethanol solution for three days. A stereomicroscope (OLYMPUS SZX16) with a maximum magnification of 20 times was used to analyze and photograph the GUS staining.

### 4.6. Cellular Localization of TaGS1;2 Using Immunofluorescence Analysis

The tissues of flag leaf, peduncle, and rachis were fixed in FAA fixative for at least 24 h. Their embedding in paraffin, sectioning, and immunofluorescence was performed by Servicebio (Wuhan Servicebio Technology Co., Ltd., Wuhan, Hubei, China). Anti-TaGS1;2 antibodies were diluted 1:200 in blocking solution. The specific antibodies of TaGS1;2 isoform was prepared in our previous study, which could monospecifically recognize TaGS1;2 subunits [17].

Negative controls were conducted by substituting the TaGS monospecific antibodies with preimmune rabbit serum (Figure S1). Microscopy detection and images were collected with fluorescent microscopy (Nikon Co., Ltd., Tokyo, Japan). DAPI glowed blue by a UV excitation wavelength of 330–380 nm and an emission wavelength of 420 nm; FITC glowed green by an excitation wavelength of 465–495 nm and an emission wavelength of 515–555 nm.

### 4.7. Extraction of Proteins from Wheat Rachis and Western Blot Analysis

Approximately 0.3 g fine homogeneous powder was mixed with 0.9 mL of GS extraction buffer (100 mM Tris, 1 mM EDTA, 1 mM MgCl_2_, 1 mM phenylmethanesulfonyl fluoride (PMSF), and 10 mM β-mercaptoethanol; pH 7.6) by shaking at 4 °C for 10 min. The extract was centrifuged at 12,000× *g* at 4 °C for 30 min. The supernatant was then prepared for further experiments. Soluble protein content was determined by the Coomassie blue dye binding method using bovine serum albumin as a standard.

Western blotting was performed in accordance with a previously described method [17]. A total of 5 μg of soluble protein extracted from the rachis was loaded onto each lane. Proteins were separated in 12.5% (*w*/*v*) polyacrylamide gel and electrophoretically transferred to 0.45 μm pore size PVDF membranes (Merck Millipore Ltd., Darmstadt, Germany) in transfer buffer (25 mM Tris-base and 192 mM Gly, 10% methanol) at 200 mA for 50 min. The membranes were blocked with TBST (20 mM Tris-base, 150 mM NaCl, and 0.05% (*v*/*v*) Tween 20; pH 7.4) containing 5% skimmed milk at 4 °C overnight. The PVDF membrane was incubated at 20 °C for 1.5 h with the TaGS1;2 antibody, and the dilution ratio of the antibody was 1:30,000. After washing three times with TBST, the membrane was incubated at room temperature for 1 h with horseradish peroxidase-conjugated goat anti-rabbit IgG (ABclonal Biotechnology Co., Ltd., Hubei, China) at 1:25,000. After several washes with TBST, the membrane was incubated at room temperature for 5 min using Omni-ECL^TM^ Femto Light Chemiluminescene Kit (EpiZyme Biotech Co., Ltd., Shanghai, China); the signals were detected by ChemiDocTM XRS^+^ Imaging System (Bio-Rad Laboratories, Lnc., Hercules, CA, USA).

### 4.8. Phloem Exudate Collection

At AS and 16, 24, and 30 DAA, the phloem exudates of flag leaves were collected according to Simpson and Dalling [44], with some modifications. Flag leaves (containing about 5 cm sheath) were removed from the plants and recut under water before rapid immersion in the collection solution (10 mM HEPES and 10 mM EDTA, adjusted to pH 7.5 with NaOH). After 15 min, the leaves were transferred to a fresh collection solution for phloem exudate collection. Three flag leaves were placed in a 1 mL collection solution in a dark and humid chamber. Exudates were collected over 4 h from 11:00 to 15:00 h Beijing time.

Phloem exudate collection of peduncles was carried out according to Wei et al. [17] with some modifications. At AS and 16, 24, and 30 DAA, the phloem exudates of peduncles on the stems were collected. The spike was cut off, and the peduncle under water was recut before rapid immersion in the collection buffer. After 15 min, the exudates from peduncle to spike were collected with a fresh collection solution. For each experiment, peduncle was placed in 1 mL collection solution in a dark and humid chamber. Exudates were collected over 4, from 12:00 to 16:00 h Beijing time.

Ammonium content was measured according to Wei et al. [36]; total ureides level was analyzed according to Collier and Tegeder [45].

### 4.9. Statistics

All data represent the mean ± standard deviation (SD) of six biological replicates. The datasets were analyzed using Microsoft Excel (2016, Microsoft, Redmond, WA, USA). Data were statistically analyzed using SPSS version 13.0 (IBM, Chicago, IL, USA). One-way analysis of variance (ANOVA) with a Duncan post-hoc test was used to test statistical differences.

## Supplementary Materials

The following are available online at www.mdpi.com/article/10.3390/ijms222011083/s1.

## Abbreviations

GS	glutamine synthetase
N	nitrogen
NH_4_^+^	ammonium
NO_3_^−^	nitrate
NUE	nitrogen use efficiency
ALN	allantoinas allantoinase
AAH	allantoate amidohydrolase
XDH	xanthine dehydrogenase
UOX	urate oxidase
QTL	quantitative trait locus
