# Supplementary material for: A New Perspective on the Role of Glutamine Synthetase in Nitrogen Remobilization in Wheat (Triticum aestivum L.)
Source: Int J Mol Sci. 2021 Oct 14;22(20):11083. doi: 10.3390/ijms222011083 (PMC8539157; doi:10.3390/ijms222011083)
Supplement: Supplementary file 1 [file ijms-22-11083-s001.zip › Supplemental/Suppl. Figure S1-2.pdf]

Flag leaf

Peduncle

Rachis

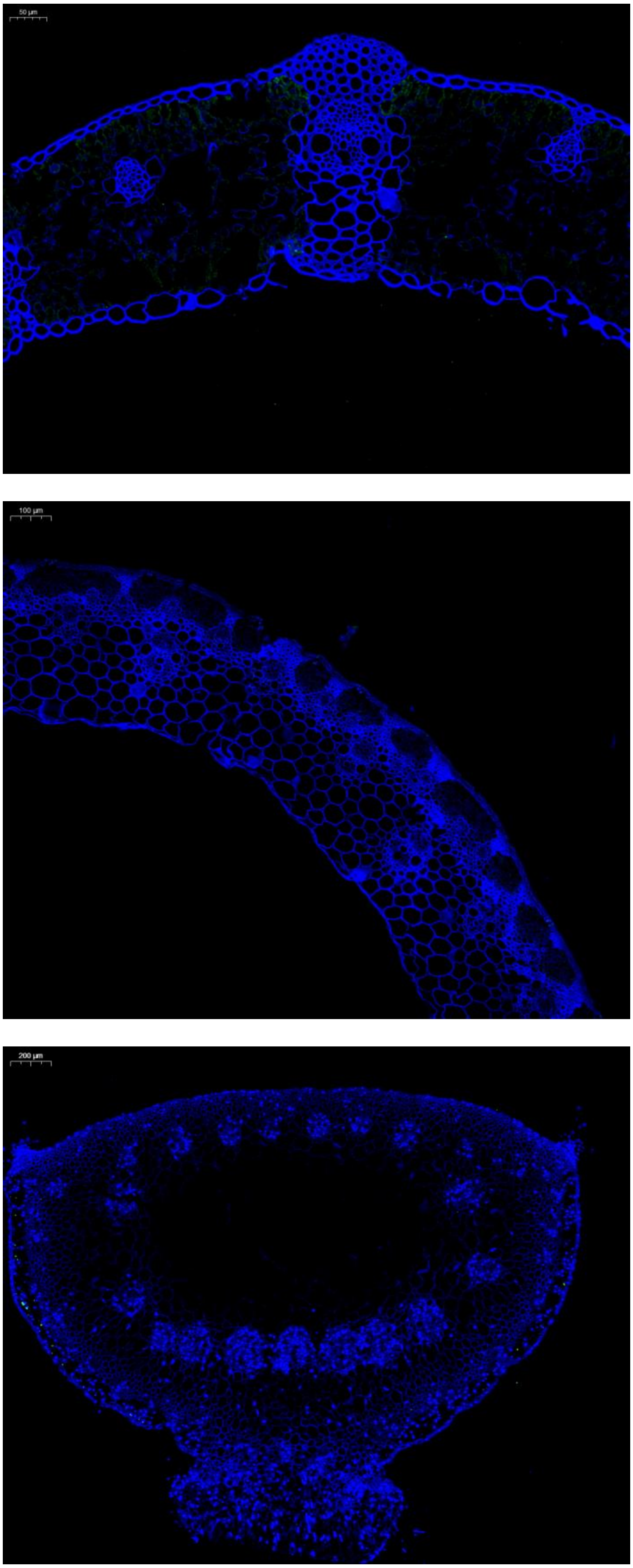

Figure S1. Negative controls section of flag leaf, peduncle, and rachis treated with pre-immune rabbit serum.

(a)

(b)

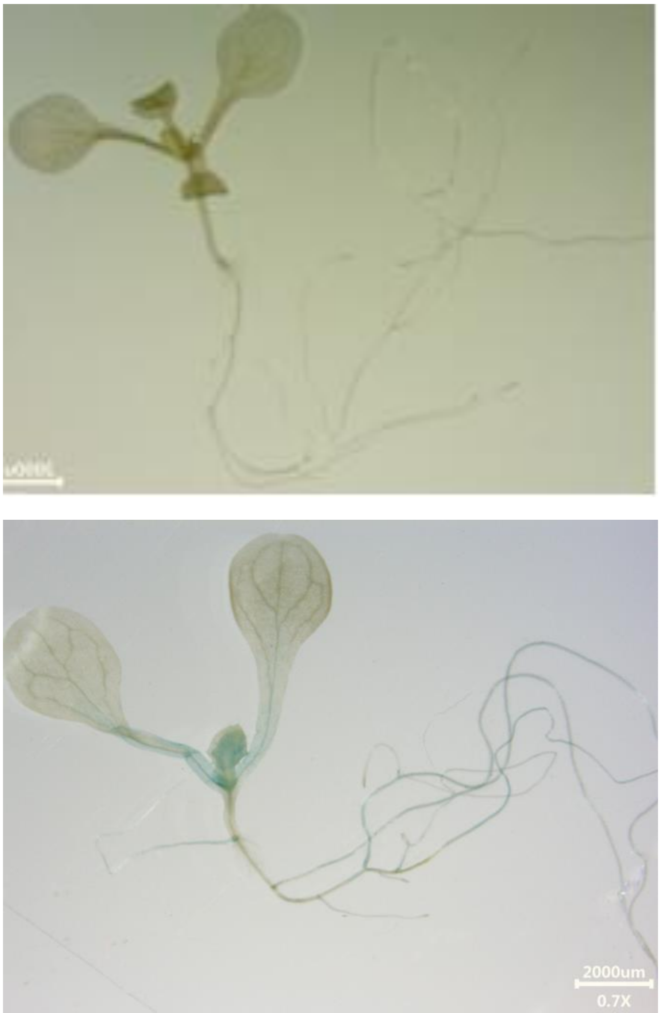

Figure S2. GUS staining of *ProTaGS2-2A::GUS* transgenic plant after 1h of natural lightinduction. (**a**) Seedling is not induced by natural light, (**b**) seedling is induced by naturallight for 1 h.
