# Supplementary material for: A New Perspective on the Role of Glutamine Synthetase in Nitrogen Remobilization in Wheat (Triticum aestivum L.)
Source: Int J Mol Sci. 2021 Oct 14;22(20):11083. doi: 10.3390/ijms222011083 (PMC8539157; doi:10.3390/ijms222011083)
Supplement: Supplementary file 1 [file ijms-22-11083-s001.zip › Supplemental/Suppl. Table S1-4.pdf]

Table S1. Meteorological data of growth seasons on air temperature and rainfall.

Month	Average air temperature (℃)	Rainfall (mm)
Oct-2019	17.2	78.9
Nov-2019	10.9	11.1
Dec-2019	2.6	23.8
Jan-2020	1.8	56.9
Feb-2020	6.3	35.4
Mar-2020	11.4	50.33
Apr-2020	15.6	19.2
May-2020	24.2	45.2
June-2020	26.6	205.8

Table S2. Primers for qPCR analysis.

Gene Name	Primer	Sequence(5'-3')
TaGS1;1-6A	*TaGS1;1-6A-F*	GTTTGGTTTGCCTGTAGCTCC
	TaGS1;1-R	CCTGAGATCCATGCCAGATC
TaGS1;1-6B	*TaGS1;1-6B-F*	CTTTCTGAGAGAAAGCTGGTGG
	TaGS1;1-R	CCTGAGATCCATGCCAGATC
TaGS1;1-6D	*TaGS1;1-6D-F*	ACGGGTTTGGTTTGCCTG
	TaGS1;1-R	CCTGAGATCCATGCCAGATC
TaGS1;2-4A	*TaGS1;2-4A-F*	ATCATTGCCATTCCCTCCTTG
	TaGS1;2-R	ATGGGGCCGTTCACCGT
TaGS1;2-4B	*TaGS1;2-4B-F*	CAGCCGGCTTCGCGC
	TaGS1;2-R	ATGGGGCCGTTCACCGT
TaGS1;2-4D	*TaGS1;2-4D-F*	GTCTTCCCCCCATTGCCA
	TaGS1;2-R	ATGGGGCCGTTCACCGT
TaGS1;3-4A	*TaGS1;3-4A-F*	CTCCCTCCGCTTGCTCTG
	TaGS1;3-R	GCTTTGCTCCTGACGTCCA
TaGS1;3-4B	*TaGS1;3-4B-F*	TACCATTTGCCACTACAACACCT
	TaGS1;3-R	GCTTTGCTCCTGACGTCCA
TaGS1;3-4D	*TaGS1;3-4D-F*	TCCCTTGCTCTGCCCTCTC
	TaGS1;3-R	GCTTTGCTCCTGACGTCCA
TaGS2-2A	*TaGS2-2A-F*	GCGTCCTTGTCCGTCTCTCT
	TaGS2-R	ACGCCCCACACCCTGC
TaGS2-2B	*TaGS2-2B-F*	CACCGGCCTCCGCTTC
	TaGS2-R	ACGCCCCACACCCTGC
TaGS2-2D	*TaGS2-2D-F*	TTGGAGGCGGCAGAGTACC
	TaGS2-R	ACGCCCCACACCCTGC
TaATPase	*ATPase-S*	CGAGGCCACCAATGACG
	ATPase-A	AGTATGGTTTCAAGAAGGCGTC
TaTEF1	*TaTEF1-S*	TCGTGGTCATTGGCCACG
	TaTEF1-A	CAGCACAGTCAGCCTGGGAG
TaXDH1	*TaXDH1-F*	ACAGTGAAAATCGTTGGAGGA
	TaXDH1-R	GCAACTTGGGCTATCTTTGTG
TaUOX	*TaUOX-F*	GTGAAGAAGTCTGGAAGCC
	TaUOX-R	CAGGAAGAAGAGTGTAGCG
TaALN	*TaALN-F*	TCGTTTCAAGTGCTCTCC
	TaALN-R	AGTTGCCTTCCTCCATTAG
TaAAH	*TaAAH-F*	TCTCTGCTCTGAAGGTCTTG
	TaAAH-R	CTCCTCGTCGCTGAATGC
GUS	*GUS-F*	GCATGTCGCGCAAGACTGTA
	GUS-R	CGACGCGAAGCGGGTAG
AtUBQ11	*AtUBQ11-F*	CGACCGAGTCGTCCTCGTC
	AtUBQ11-R	GTGTCAGAGCTTTCCACCTCG

Table S3. Primers used to amplify the promoters of TaGS genes.

Gene Name	Primer	Sequence(5'-3')	Product size (bp)	Tm (℃)
proTaGS1;1-6A	*proTaGS1;1-6A-F*	CACTGCCTTCTCAGGCTTGTTA	2000	58
	proTaGS1;1-6A-R	GCCCCATGAATTTAGCATCG		
proTaGS1;1-6B	*proTaGS1;1-6B-F*	TCTTAGTCCAACGTGGCATCATAGG	2033	62
	proTaGS1;1-6B-R	AGACGCCAAACTACGACTACG		
proTaGS1;1-6D	*proTaGS1;1-6D-F*	CACATTCACACGTGGTTTCCTCT	2261	58
	proTaGS1;1-6D-R	GCAAAAACCTCTTCTCGTCGC		
proTaGS1;2-4A	*proTaGS1;2-4A-F*	GTAATGTGGCTACGGTGTGAGTCTGTT	2155	61
	proTaGS1;2-4A-R	GGGTGCAGAATGCAAGTAGC		
proTaGS1;2-4B	*proTaGS1;2-4B-F*	TCATCTCCGTGAGGAGGTCTTGA	2308	62
	proTaGS1;2-4B-R	TACTCGACGATGATCTTGTCGGTG		
proTaGS1;2-4D	*proTaGS1;2-4D-F*	GCCCGTTGTACCACTGTTATCGAT	1624	62
	proTaGS1;2-4D-R	TACTCGACGATGATCTTGTCGGTG		
proTaGS1;3-4A	*proTaGS1;3-4A-F*	TCACCCATAATCATGCTGTGCAAAT	2538	63
	proTaGS1;3-4A-R	TACCATATGTACTCGGCGATGATCTTG		
proTaGS1;3-4B	*proTaGS1;3-4B-F*	ATGGGAGGTTGGGACAGTGC	2508	58
	proTaGS1;3-4B-R	GATGCGAAGCATACGCGC		
proTaGS1;3-4D	*proTaGS1;3-4D-F*	TCTGAAGATGCTCATGAGTTGCTCAAT	2536	62
	proTaGS1;3-4D-R	TACCATATGTACTCGGCGATGATCTTG		
proTaGS2-2A	*proTaGS2-2A-F*	GCATTCCTGGCTTGCATGTCTAGA	2008	58
	proTaGS2-2A-R	ATGATCTTGTCGGTGAAGGGC		
proTaGS2-2B	*proTaGS2-2B-F*	ATTTCGCCGAGTTCAGGTCGAG	2153	63
	proTaGS2-2B-R	ATGATCTTGTCGGTGAAGGGC		
proTaGS2-2D	*proTaGS2-2D-F*	CAGCGATCCTAGCCTTCGAAAAG	1878	60
	proTaGS2-2D-R	ATGATCTTGTCGGTGAAGGGC		

Table S4. Primers used to construct the *ProTaGS:GUS* fusion vector.

Gene Name	Primer	Sequence(5'-3')
proTaGS1;1-6A	*proTaGS1;1-6A-F*	CAGCTATGACCATGATTACGAATTCCCGCTCTAAACCCTGACCCC
	proTaGS1;1-6A-R	TTACCCTCAGATCTACCATGGGGCGGGAGGAGCAATCC
proTaGS1;1-6B	*proTaGS1;1-6B-F*	CAGCTATGACCATGATTACGAATTCGCATCATAGGCACCCGCC
	proTaGS1;1-6B-R	TTACCCTCAGATCTACCATGG GGCCGGCTGACTGAGGG
proTaGS1;1-6D	*proTaGS1;1-6D-F*	CAGCTATGACCATGATTACGAATTCCACATTCACACGTGGTTTCCTCT
	proTaGS1;1-6D-R	TTACCCTCAGATCTACCATGGGGCCGGCTGACTGAGGG
proTaGS1;2-4A	*proTaGS1;2-4A-F*	CAGCTATGACCATGATTACGAATTCAGCAAACCTAGACCTCATCCCAG
	proTaGS1;2-4A-R	TTACCCTCAGATCTACCATGGTGCTGCTGCCGGTGCTACT
proTaGS1;2-4B	*proTaGS1;2-4B-F*	CAGCTATGACCATGATTACGAATTCTTGAAAGCTGCAGGGGCC
	proTaGS1;2-4B-R	TTACCCTCAGATCTACCATGGTGCTGCTGCTACTCGCAAGG
proTaGS1;2-4D	*proTaGS1;2-4D-F*	CAGCTATGACCATGATTACGAATTCGCCCGTTGTACCACTGTTATCGATTC
	proTaGS1;2-4D-R	TTACCCTCAGATCTACCATGGTGCTGCTGCTACTACTCGCAAGGAG
proTaGS1;3-4A	*proTaGS1;3-4A-F*	CAGCTATGACCATGATTACGAATTCATGGCTTCGAGACATGTGGTT
	proTaGS1;3-4A-R	TTACCCTCAGATCTACCATGGGGCTGCTACTTCTTCTTCTTCTTCC
proTaGS1;3-4B	*proTaGS1;3-4B-F*	CAGCTATGACCATGATTACGAATTCGCGCTGGGATGGGCTGGAGC
	proTaGS1;3-4B-R	TTACCCTCAGATCTACCATGGGGCGGATGCCGGCGTGTC
proTaGS1;3-4D	*proTaGS1;3-4D-F*	CAGCTATGACCATGATTACGAATTCGGCATGGATATCGTTGTAGAGGC
	proTaGS1;3-4D-R	TTACCCTCAGATCTACCATGGGGCTACCTCTTCTTCTTCTTCTTCCC
proTaGS2-2A	*proTaGS2-2A-F*	CAGCTATGACCATGATTACGAATTCAGGAACTGTGGCTTCGAGGAT
	proTaGS2-2A-R	TTACCCTCAGATCTACCATGGCGCCGCTACTTACTTACTTACAGGT
proTaGS2-2B	*proTaGS2-2B-F*	CAGCTATGACCATGATTACGAATTCTCTAATTTCCGGATCAGTTCTTGTA
	proTaGS2-2B-R	TTACCCTCAGATCTACCATGGCGCCGCTGCTTGCTTACT
proTaGS2-2D	*proTaGS2-2D-F*	CAGCTATGACCATGATTACGAATTCAGGAACTGTGGCTTCGAGGAT
	proTaGS2-2D-R	TTACCCTCAGATCTACCATGGCGCCGCTACTTACTTACTTACAGGT
